# Community Building in Crisis: Interventions With Displaced Communities in Temporary Housing Shelters

**DOI:** 10.1002/jcop.23170

**Published:** 2024-12-23

**Authors:** Iris Posklinsky, Hani Nouman

**Affiliations:** ^1^ School of Social Work University of Haifa Haifa Israel

**Keywords:** community building, community intervention in disasters, community psychology, displacement, social capital

## Abstract

Emergency displacement is a widespread phenomenon impacting all facets of life and increasing rates of psychological trauma. This article analyzes the effects of shared residency among evacuated and fragmented communities, focusing on social and communal disparities in temporary shelters. By adopting theoretical perspectives of community building and social capital, it describes interventions aimed at promoting resilience within newly formed shelter communities. A qualitative study was conducted in temporary housing shelters in Israel during wartime. The study included four focus groups with 31 professionals and an analysis of recent emergency protocols. Professional interventions following displacement into temporary evacuation centers create a new reference community, “the shelter community.” These mobilizing interventions include establishing volunteer emergency teams, strengthening community cohesion, and fostering community participation in renewed daily routines. Community building interventions in temporary housing shelters can foster personal and community empowerment following disasters worldwide.

## 1 Introduction

Displacement following emergencies is a growing global phenomenon. Natural disasters, war, and terrorism are increasing while the number of evacuated people continues to expand. By the end of 2022, their estimated number reached a record high of 71 million individuals (Internal Displacement Monitoring Centre 2023). Displacement is often described as a traumatic experience with profound psychosocial ramifications. The uncertainty of leaving home for an unknown period, combined with disrupted routines in all aspects of life, fundamentally erode feelings of security, stability, and control (Huang and Wong 2013; Norris et al. 2008; Oldfield and Stevenson 2024; White and Van der Boor 2021).

Upon evacuation, displaced populations are often offered temporary housing solutions, which may include sheltering in public facilities, hotels and lodgings, caravan neighborhoods, and other mobile or permanent housing structures (Kromm and Sturgis 2008; Mitchell, Esnard, and Sapat 2012). While related literature offers various definitions of temporary housing (Johnson 2002; Quarantelli 1995), displacement from one's permanent home to an alternative residence for up to 3 years is generally considered a temporary living arrangement (Félix, Branco, and Feio 2013). However, temporary housing can be more than just a physical space. This study illustrates the actions and dynamics associated with a community building process within temporary housing, culminating in the formation of a temporary community. A temporary community within shelters emerges as residents, bound by shared traumatic experiences that led to their evacuation, come together to engage in collective actions, develop and maintain social networks, and foster resilience by empowering the community as a whole (Wilson 2013).

Post‐disaster temporary housing is essential not only for establishing a sense of protection and normalcy in the lives of evacuated communities (Félix, Branco, and Feio 2013) but also as an environment where social capital and active community participation are fostered (Aldrich 2011). Vulnerable populations, such as the elderly and the poor, are less likely to possess the individual social capital and resilience necessary to cope effectively with a disaster, or secure privately‐arranged housing solutions (Aldrich and Meyer 2015; Ma et al. 2013). In this context, temporary housing can serve as a space for enhancing social capital and building communal life among the displaced. Koyama et al. (2014) found that residing in temporary housing was a predictor for accessing social support, while Félix, Branco and Feio (2013) asserts that recovery is achieved through the reestablishment of communal life in post‐disaster temporary housing. A comprehensive body of empirical studies on displacement shows that positive recovery outcomes for evacuated communities are closely linked to their ability to maintain community support systems (Benhura and Naidu 2021; Kuroda and Koyama 2020; Ludin, Rohaizat, and Arbon 2019; Ma et al. 2013), levels of trust in neighbors and relatives (Lee and Kim 2021), sense of community and shared identity (Chamlee‐Wright and Storr 2011), and levels of participation in community activities (Rayamajhee and Bohara 2021).

Displaced communities that remain intact in temporary housing solutions that preserve their social continuity tend to recover and build resilience more effectively (Aldrich 2011; Benhura and Naidu 2021; Ma et al. 2013). However, post‐disaster evacuation is predominantly an immediate emergency action aimed at mitigating imminent threats to life. As an urgent response, it may result in relocating an entire organic community to a single temporary shelter, or in fragmented communities that are dispersed across multiple shelter facilities as dictated by emergency constraints. Consequently, temporary housing centers may house in close proximity diverse demographic groups with cultural, religious, or socioeconomic disparities. The literature elucidates that disasters impact the diversity and density of networks and social capital due to the resettlement of displaced populations (Dussaillant and Guzmán 2014). This demographic heterogeneity may engender interpersonal tensions and “us versus them” behaviors, hindering individuals’ capacity to develop bridging ties with members of different communities (Agnitsch, Flora, and Ryan 2006; Lee 2020).

Community interventions in temporary housing shelters accommodating displaced and fragmented communities may prove instrumental in enhancing community resilience. They address the unique needs of emergency resettlement, including mobilizing resources, social capital, and networks within and across diverse communities (Adger et al. 2005; Scannell et al. 2016). To the best of our knowledge, community building interventions in temporary housing shelters among split and fragmented communities remain largely unexplored. In this study, we aim to empirically address this gap in academic research.

### 1.1 Community Building and Social Capital in Crisis

In the absence of a community structure among populations sharing a common space, community building practices establish social structures that foster a sense of community and help develop it. The situation of emergency displacement to shelters exemplifies a scenario where individuals from diverse backgrounds converge in a new environment, compelled to live under temporary and uncertain conditions. The disruption of life routines, along with its ramifications, necessitates a reorganization. However, before a community can be reorganized, it must first be built if one does not already exist. Rather than emphasizing problem‐solving, the practice of community building primarily concentrates on the community itself and developing its capacities (Minkler 2012). Community building promotes readiness to reorganize community life, creating an environment where shared problems can be effectively addressed. The process strives to identify, develop, and activate local resources to meet the unique needs of the community (Chaskin et al. 2006).

Community building focuses on the social unit as an inclusive, multidimensional, and dynamic system, regarding the community worker as an integral part (Walter and Hyde 2012). Among other roles, the professional enhances resident participation and engagement, identifies leadership and resources, develops and activates them (Blackwell and Colmenar 2012; Rothman 2007; Weil 1996). These functions are associated with establishing community structures and infrastructures through which residents can influence shared goals. In a chaotic setting, professionals, who understand the needs of vulnerable populations and are experienced in navigating complexities embedded within community networks can play a critical role in helping them recover (Yanay and Benjamin 2005). Following a disaster, emergency interventions focusing on community building are among the most effective responses to mobilize social change and promote community resilience (Pyles 2007).

Community resilience refers to the capacity of individuals to mobilize forces and resources through strategies of cohesion and community organization. It involves working together to address shared goals and overcoming complex situations or collective trauma (Ganor and Ben‐Lavy 2002‐2003; Pfefferbaum, Pfefferbaum, and Van Horn 2015; Tavares et al. 2023). Community resilience positively influences its ability to cope with prolonged stressful situations (Kulig et al. 2013; Perdomo, Sánchez, and Blanco 2021). In dealing with disasters, community building can lead to community resilience by strengthening communal social capital. In this process, “resources embedded in a social structure… are accessed and/or mobilized in purposive action” (Lin 2001, p. 29). A community with strong social capital will demonstrate mutual responsibility and support that are not necessarily dependent on external forces. It will be able to care for its vulnerable groups from within (Carmen et al. 2022; Guo and Li 2016). Thus, the interplay between community building, social capital, and community resilience forms a crucial framework for understanding and addressing the challenges faced by displaced communities in temporary housing shelters.

Community interventions that strengthen social capital among displaced individuals are implemented along with community building processes. They are crucial for reinforcing social support networks and establishing the infrastructures and processes necessary for shared life after a disaster (e.g., Aldrich and Meyer 2015; Scannell et al. 2016; Ludin, Rohaizat, and Arbon 2019; Ma et al. 2013). These interventions acknowledge the diversity within the community and strive to cultivate competencies in the entire community system and among all its participants. Residents can collaboratively engage in finding solutions that strengthen community resilience. For example, residents may act as local leaders involved in establishing social structures and formal and informal networks, they can participate in assessing concerns in their communities, engage in collective thinking about possible solutions, or take collective action for change (Douglas et al. 2016; Zimmerman 2000). Research indicates that active displaced residents who are organized around a collective, hope‐oriented task exhibit enhanced personal and community self‐efficacy (Hobfoll et al. 2007). When people who are closest to the problem become part of its solution, they feel empowered and capable (Christens, Inzeo, and Faust 2014; Stahlhut and Bowes 2003).

### 1.2 Disparities in Social Capital and the Necessity for Community Building

In temporary housing shelters, displaced and fragmented communities with cultural, religious, or socioeconomic diversity may cohabit in close proximity. They may differ in their social capital, demonstrating variations in their capacity to navigate crises as a cohesive community. Social capital disparities may be seen in differences in relationships, social networks, action outcomes, resources, and support among community members (Aldrich and Meyer 2015). Therefore, social capital gaps have substantial ramifications for community building processes. When individuals, families, and communities residing together in temporary shelters lack shared norms and identities, it is essential to implement interventions that strengthen community capital and cohesion among its members. Such interventions aim to reinforce commonalities while fostering engagement and collective organization of residents in dealing with the emergency situation (Binder et al. 2023; Meyer 2018; Ritchie and Gill 2018). In this context, Hobfoll et al. (2007) posit that disaster survivors often share a sense of common fate, encapsulated in the notion of “swimming or sinking together” (p. 294), which provides a framework for their collective action.

Disparities in social capital may also stem from attachment of displaced individuals to their original place‐based community. Place attachment represents solidarity with community members, concern for the community's future, and commitment to shared objectives among its residents (Ilovan and Markuszewska 2022). It spurs participation in local action and leadership, enabling the community to confront disasters together. It involves a culture of caring neighborliness (Scannell et al. 2016), strengthening community belonging and bonding social capital (Low and Altman 1992). Consequently, a community with strong place attachment may exclude those not considered an organic part of it (Ilovan and Markuszewska 2022). When displaced individuals with strong ties to their original communities converge with others in temporary housing centers, exclusion between closed community groups may emerge, potentially challenging the process of building a new, cohesive community. Therefore, clarifying community interventions that overcome social capital disparities and enhance community resilience through building a new community is crucial.

The literature has extensively dealt with the psychosocial ramifications of displacement and interventions that assist displaced communities (Oldfield and Stevenson 2024; Maya‐Jariego et al. 2019; White and Van der Boor 2021; Perdomo, Sánchez, and Blanco 2021). However, there is a dearth of empirical evidence regarding interventions in temporary housing shelters, particularly concerning community building among displaced and fragmented communities. An in‐depth focus on communal interventions in temporary housing shelters can enhance the capacity of professionals to strengthen community resilience in an era of widespread and frequent disasters.

## 2 Methods

### 2.1 Context of the Current Research

To delineate the challenges and professional interventions with displaced fragmented communities in temporary housing shelters, a qualitative study was conducted in Israel during wartime. More than a quarter of a million residents were evacuated from dozens of localities across two combat fronts in Israel, along the Lebanese border in the north and the Gaza border in the south. The civilian evacuation was prompted by an unexpected terrorist attack on October 7, 2023. During the attack, approximately 3000 terrorists infiltrated from Gaza into Israel, executing a mass atrocity that resulted in 1117 fatalities across 38 southern towns and villages, including 366 participants in a music festival. Additionally, 257 individuals were abducted into Gaza, against a backdrop of extensive rocket fire directed at Israel (Elron, Shapira, and Deitch 2024; Lerer 2023; The Institute for National Security Studies 2024).

These displaced individuals were relocated to temporary housing in 434 shelter facilities in hostels, hotels, and dormitories (Government decision No. 975 & 988 n.d.; Zidenberg‐Gochman and Shabtai‐Gatehon 2023). The majority of the localities suffered substantial casualties, both physical and psychological. Ilovan and Markuszewska (2022) term the impact of such destruction as a “soul‐destroying effect”. They refer to a traumatic experience of displacement from home that is accompanied by loss and grief over forced changes, which disrupt and destroy living environment. This unprecedented situation necessitated rapid adaptation of existing infrastructure to meet the immediate needs of the displaced population.

In this reality, the support of professionals in community building was essential to empower vulnerable populations and promote community resilience. For this reason, it was crucial to identify the challenges faced by displaced and fragmented communities, as well as the ways in which professionals assisted these communities. This knowledge is pertinent for communities worldwide that may be evacuated to temporary housing shelters in crisis and disaster situations. Therefore, this research contributes to the growing body of knowledge of disaster‐induced displacement and community resilience, offering insights that are applicable to diverse and global contexts.

### 2.2 Data Collection

#### 2.2.1 Focus Groups

“Data collection is the basis of any successful empirical study” (Small and Calarco 2022; p. 145). This qualitative research aimed to collect the most relevant real‐time data on community building efforts in shelters directly from professionals actively engaged in the field. In December 2023, both authors conducted four focus groups via Zoom. The Zoom platform was selected to facilitate nationwide participation, ensuring a diverse geographical representation. A total of 31 professionals participated in these groups, including 21 social workers who operated in temporary housing shelters across the country and 10 social workers in senior management positions in the Ministry of Welfare who were responsible for knowledge development, policy implementation, and support of front‐line social workers operating in shelters. Each group discussion lasted 90 min, centering around the experience of professionals as they provided aid to displaced communities and the methods they employed to do so. Groups were facilitated by the researchers and followed an interview guide to stimulate a focused discussion. Some of the questions were: What are the challenges faced by displaced and fragmented communities in the temporary housing shelters? What communal interventions were undertaken by professionals to strengthen community capital and promote community resilience among displaced communities?

Our approach emphasized sensitivity to the research context and self‐awareness of the unique circumstances faced by our participants, who were working and, in some cases, living in shelters. As suggested by Small and Calarco (2022), researchers’ self‐awareness promotes empirical effectiveness, particularly when applied before, during, and after the Interviews. In our study, we engaged in reflective dialog before and after each focus group, carefully attended to participant disclosures and state of being during sessions, and ensured their authenticity is well‐represented in our data analysis and interpretation.

The dialog between senior management and frontline social workers in joint focus groups enabled a two‐way discourse of learning, exposing challenges and coping with the complexity of providing response in hundreds of temporary housing shelters. All focus groups were audio‐recorded, transcribed, and subsequently subjected to rigorous thematic analysis.

#### 2.2.2 Textual Sources

The findings from the focus groups were integrated with information from complementary textual sources. These primarily included guidelines and regulations developed by the Ministry of Welfare from the outbreak of the war in early October 2023, as well as protocols for professional interventions in temporary housing shelters. The objectives of the document analysis were to deepen the understanding of work methods against the backdrop of government policy and to become familiar with work concepts developed in shelters.

#### 2.2.3 Analytical Approach

In this qualitative research, an inductive approach was used (Charmaz 2014) to extract from the accumulated knowledge central insights about challenges and professional interventions with displaced fragmented communities. During data analysis, validity of qualitative research is enhanced by avoiding the imposition of pre‐conceived categories on the data and committing to transparency and coherence, clearly showing how conclusions are derived from the data (Yardley 2017).

Our analysis process included several stages: First, texts from focus groups and document sources were read and analyzed. Open coding was performed for the content units that emerged from the findings. Secondly, concepts and categories derived from each content unit were examined while unifying several categories into a theme. Then, axial coding was used to compare and connect categories and to identify cross‐cutting themes across different knowledge sources. The analysis continued until no new content was generated (Green and Thorogood 2018). Both authors led the data analysis while maintaining an ongoing discourse between them. The purpose of the dialog was to examine the data and its relevance to the research objectives. This focused lens allowed for clear definition of the themes that emerged from the analyses and to support them with relevant quotes. The inductive approach allowed for the emergence of nuanced insights that might have been overlooked in a more deductive, hypothesis‐driven analysis. Insights generated from the data analysis are displayed alongside participants voices to illustrate the findings.

#### 2.2.4 Research Ethics

The research was approved by the Institutional Review Board at the university of the two authors. Rigorous adherence to ethical protocols was maintained throughout all stages of the research. Participation was voluntary. Participants were explained that the purpose was to extract knowledge and insights from the field and all identifying information would be removed. To protect the identity of participants and the data provided, their identifying information was separated from the content reported in the article. All data were stored securely in encrypted formats, with access restricted to the primary investigators.

## 3 Findings

The research findings are organized according to two central themes: (a) challenges in temporary housing shelters and (b) intervention principles for building communities in temporary housing facilities.

### 3.1 Challenges in Temporary Housing Shelters

Community building during crises is complex due to the elevated psychological distress experienced by residents and professionals who are exposed to trauma. The multiplicity of challenges is further amplified when the community is uprooted from its natural place to a temporary environment, cohabiting with other evacuees and unfamiliar communities, for an unknown period. Analysis of the findings revealed two main challenges: weakened social capital against the backdrop of social heterogeneity, and difficulty in maintaining positive community momentum over time.

#### 3.1.1 Weakened Social Capital in Fragmented Displaced Communities

When social gaps and diversity were high among displaced communities, the ability of professionals to enhance social capital was particularly challenging. Social diversity was manifested in cultural, religious, socioeconomic backgrounds, and even political views. Diversity was also evident in differences between cohesive rural settlements versus dispersed urban neighborhoods, as well as the representation of minority communities versus majority. The intense reality of shared housing in shelters magnifies tensions associated with gaps between evacuated communities, and augments perceptions of class differences present in routine times. Several professionals described incidents of frustration, resistance, and uprising: “*The diversity of the community and the varied populations led to tensions and conflicts*”; “*Feelings associated with marginalization and inequality intensified and often led to mutual accusations and anger. Just one sentence said about someone from a certain place—could shape the image of their entire community*” (FG 1).

Rural communities, particularly kibbutzim (small communal settlements), characterized by active and strong social capital, normally operated community emergency teams and maintained rich community life as a routine. Upon their evacuation, they naturally and quite immediately managed to organize their people in the housing shelters. They established structures to mobilize their community, created responses to a variety of needs among their members, and cultivated life routines and community meetings that increased the sense of solidarity between them. Urban populations unaccustomed to community life and experiencing lower socioeconomic status, were also evacuated to housing shelters, often sharing space with evacuees from Kibbutzim. Connecting and bridging these different communities posed a challenge in developing social capital and building an integrated shelter community.

In light of this, the professionals who participated in the study emphasized the need to create opportunities for mutual dialog that challenges prejudices, enables mutual acquaintance between groups, and strives to discover shared values. The professionals saw themselves committed to the task of bridging between the diverse groups, as one participant said: “*When I'm there, things become clearer*” (FG 3). Another participant explained that they “*engage in mediations and strive to achieve agreements… when you confront situations with concrete actions and statements ‐ everything that sits on prejudices, on different values, suddenly comes down to reality… the masks fall*” (FG 1). Participants testified to their need as professionals to be active in maintaining connections between the displaced, stating that “*to maintain the hotel community, we need to hold it together, to create the glue*” (FG 4).

#### 3.1.2 Maintaining Positive Community Momentum in Temporary Housing Shelters

Paradoxically, displacement into a shared housing shelter offered some positive aspects, especially for those who had lived in isolation during routine times and for those who had never experienced the benefits of a community. Elderly individuals who were previously confined to solitude found themselves embraced by a supportive community in the shelter. Acknowledging that, one of the participants wondered “*how can we prevent a crisis upon returning to loneliness in the neighborhoods, especially now after they confronted terrorism and severe trauma?*” (FG 4). Other evacuees experienced empowerment and discovered capabilities through their involvement in community building activities. Communal professionals and other service providers in the facility fostered a supportive environment where the actions undertaken by the evacuees were encouraged. They learned to reach out and network within a complex system, work in teams, and solve problems as a community. During the first few weeks of the evacuation, the motivation of volunteers was noticeable; however, over time, motivation diminished, necessitating to train new volunteers to fulfill different roles in the community.

Evidently, burnout emerged early in the communal living arrangement, likely triggered by the high relational density associated with life in shelters. Many residents grew weary of the close proximity of communal life, stating, *“Normally I'm a very community‐oriented person… I understand that communities are strong when they're together, but we're tired of being together*” (FG 4). Discussions evolved around the challenge of “*maintaining the high operational tension needed for emergency response…*” and “*the need to constantly hold and maintain the operation*”. Burnout was caused not only by the intensity of life in shelter and the crowded facility but also by blurring boundaries between private and public spheres. In this sense, close community may also be experienced as a burden. Counseling took place in the hotel lobby, and couples had to talk and sometimes argue without any intimacy. That sense of burden led to a suggestion made by one of the participants to “*create virtual community spaces where the displaced can meet. It may reduce crowding in the hotel*” (FG 4).

Considering the above, a high turnover of displaced persons in temporary housing shelters became apparent after a short period. Stronger populations were the first to leave and secure private rental options. These departures affected the community climate, while those left behind often lacked the personal capacity to be displaced again. Particularly when active leaders left, it undermined the trust and sense of security of those remaining: “*It was very dynamic… people moved to other places and there was no stability. Those who left were no longer leaders in the community*” (FG 3).

### 3.2 Intervention Principles for Community Building in Temporary Housing Shelters

The communal intervention principles in shelters, particularly in the initial weeks following the evacuation, evolved in an acute crisis environment while mitigating to some extend the sense of provisionality. The intense reality necessitated flexible, agility, resourcefulness, and problem‐focused approach. As evacuees settled into shelters and their basic needs were met, the process of community building began to emerge, centering around three core strategies: (a) establishing emergency shelter teams based on local leadership; (b) strengthening connections within and between communities to promote social cohesion; and (c) activating the community around new and renewed life routines.

#### 3.2.1 Establishing Volunteer Emergency Teams in Shelters

A central intervention principle for community building in temporary housing shelters is the establishment of an emergency team comprising local leaders who represented the various communities. Some leaders were active in their home communities before the evacuation, while for others, this was their first experience as volunteer leaders. The teams were so vital that one of the participants noted: “*The community emergency teams we built were the first to start working. They're the ones who got up and knew what to do in the hotel… Without them, reality would have looked different*” (FG 4). This intervention enhanced the capability of displaced communities by virtue of participation in community action. Being experts in their own needs, it encouraged them to take responsibility for articulating solutions. “*They are the ones who could engage the community. Those who weren't scared by the chaos and worked directly with the rest of the evacuees, and didn't fall silent ‐ they marked the difference between functioning and nonfunctioning*” (FG 4).

The primary purpose and role of the emergency teams were to serve as an immediate and central point of contact for the many needs experienced by the displaced: “*They're the ones who took care of the most basic things ‐ changing hotel rooms, dealing with financial needs of residents, and identifying safety issues*” (FG 4). Some of these needs were brought to the attention of professionals on‐site, while others were managed independently by the volunteers. Professionals reported various types of needs arising in the shelter, including basic necessities due to hasty home abandonment, clean clothes, a washing machine and organizing its usage schedule among hundreds of other evacuees, youth wandering at night, and medication supply. It was evident that social and personal problems not only didn't disappear with the evacuation but even worsened in some cases. Therefore, psychosocial needs such as domestic violence, drug use, sexual abuse, and loneliness also required professional's attention in the shelter.

The emergency teams were divided into taskforces or functions with distinct areas of responsibility, such as logistics, communication, social activities, and medical response. Each team was headed by a volunteer coordinator who, alongside the community worker, led the day‐to‐day management of the displaced community in the shelter. The coordinator recruited and staffed roles to carry out tasks, networked with external agents, and generally instilled an atmosphere of control and coping with the situation. The work of the shelter emergency teams was agile and evolved according to changing needs.

Managing the emergency teams involved overseeing their composition, defining roles, and establishing decision‐making processes. To reach agreements and reduce tension among the displaced, there was a deliberate effort to create team with maximum representation from the evacuated communities. Typically, there was a range of populations from different cultural and religious backgrounds: “*A community that was entirely Arab Muslim… spoke Arabic all the time. It was important that they too felt a sense of togetherness, that what was happening was common to everyone*” (FG 2). On the other hand, Ultra‐Orthodox Jewish evacuees participated in community actions that were suitable for them: “*They gathered to recite prayers for peace and the recovery of the injured, they went over the names of the hostages and prayed for their well‐being, they donated food*” (FG 2). Assembling mixed teams was a task that community workers usually took upon themselves because “*not all volunteers were comfortable with that…*” (FG 4). Whether interacting with the leading team, the hotel staff, shelter residents, or external agents, building community in shelters was a highly complicated endeavor that required aligning expectations and accommodating diverse populations. Forming a strong, empowered, and qualified emergency team was an essential part of the process.

#### 3.2.2 Strengthening Social Cohesion Within and Between Communities

The organic displaced community, which was exposed to collective trauma back home, served as the primary reference point for evacuees in the shelters and constituted a significant resource for knowledge, support, and solutions to shared problems. This phenomenon aligns with the concept of collective trauma and community resilience as discussed by Norris et al. (2008). On one hand, their congregation under one roof represented a source of support and solidarity, but on the other hand, it created multiple tensions, as previously mentioned. Bridging between the various communities in the shelter and strengthening cross‐community cohesion was a central task of the professionals, performed in collaboration with the emergency teams. As one participant noted, “*Community cohesion developed over time. It wasn't there from the outset*” (FG 3).

Enhancing social cohesion in a mixed community environment included organizing inclusive dialog meetings, joint activities, and building communication channels to alleviate tensions. These efforts led evacuees to understand that “*everyone is in a similar reality and shares a common fate*” (FG 1). This approach resonates with the social identity model of collective resilience proposed by Drury et al. (2019). According to participants, actions aimed at fostering community cohesion around a shared ethos are essential. Therefore, they gathered residents for activities of “*common interests and cooperation”* (FG 3), which enhanced a sense of reciprocity and commitment to the collective.

In one of the hotel shelters, a professional described how she connected residents from different communities and built partnership between them by creating a shared narrative: “*We talked about how we connect to another community from a different town… We discussed the roles, the place of each community, of the activists, of the leaders… Later, we created a logo for the community, printed our own t‐shirts, and organized a joint run… We held a cultural evening for both communities… We worked together! It was so important to create the connection, talk, and cooperate. Only that way there can be understanding, there can be acceptance and agreements*” (FG 3). This process exemplifies the creation of a superordinate identity, a concept central to intergroup contact theory (Pettigrew and Tropp 2006).

While emergency volunteer teams operated in many of the shelters, almost all home communities retained a minimal presence of security personnel, farmers, or residents who had difficulty fleeing, or refused to leave their homes despite the security threat. The connection with the home community provided important contact and updates on the state of the evacuated localities. It allowed evacuees to gather around their shared concern for the place, the ruined properties, the community members who were hospitalized after being injured, families of the abducted residents, or community members who were still missing. This ongoing connection aligns with place attachment theory and its role in community resilience (Aldrich and Meyer 2015; Scannell et al. 2016).

In the first weeks, there was considerable tension surrounding the contact with the evacuated localities. The thirst for information on the threat to the settlement, plans for its rehabilitation, and the prospect of returning home “*was constantly on everyone's mind*…” (FG 4). Additionally, it was noted that while those left behind lost a sense of community life, evacuees in hotels experienced heightened community engagement. In cases where a strong home‐community was split between several shelters, efforts were made to create a virtual community space to preserve the connection between their members. However, it was reported that “*for those in a shared physical space, it was easier to maintain a sense of community*” (FG 4).

#### 3.2.3 Activating the Community Around New and Renewed Life Routines

The third core principle of community building interventions in temporary housing shelters focuses on establishing social structures to operate and manage life and encourage engagement in a developing community. A key structure is the emergency team, which mobilizes volunteer to lead the organization of communal life. Additional structures include assemblies and gatherings of the entire shelter community for information sharing, affiliation and therapeutic groups, and social clubs for various age groups. Volunteers and professionals held regular meetings, and weekly events were planned to provide relief and leisure.

Around these structures, activities and routines were established in the evacuation shelters, forming a vital channel for residents to function both individually and as a community. Life routines were restored in formal areas such as children's education and adults’ employment, reducing chaos and stress in the new reality. As one participant noted: “*Until children were back in school (in the refuge locality), nobody was available…*” (FG 1). Community building in shelters promoted conditions and processes that enabled the restoration of life routine, adapting to the unique situation and environment forced on displaced individuals. Routine provided a framework for functional continuity essential for recovery in shelters: “*We created a new narrative, within the new situation, and within a new community*” (FG 1).

Considering these aspects of community building strategies, it is clear they were essential for fostering interactions that sustained the community. They formed multidimensional platforms for connections and collaborations within the shelter community and with external systems. The multidimensional nature of these interfaces included cross‐ communities’ structures—connecting evacuees from different communities; cross‐role structures—linking evacuees with hotel representatives and employees; and cross‐sector structures—bridging evacuees with business organizations, public, and third sector agents. These processes enhanced the ability of the new community to cope with their situation. While many external agents expressed interest in assisting the evacuees, empowerment was crucial for their rebuilding and recovery: “ *There was a clear understanding that they are helping, not managing their life*”; “*The leadership of the shelter community is the one that made decisions. We, as professionals, only enabled the process*” (FG 3).

## 4 Discussion

Extreme situations of collective trauma are on the rise around the globe, often resulting in displacement and evacuation of entire communities from their place of home. Millions of displaced individuals face the challenge of rebuilding their lives in temporary evacuation shelters. However, to the best of our knowledge, there is a lack of studies documenting interventions for community building in these settings. Enhancing personal and collective resilience to cope with disasters and displacement requires developing community capacities and taking actions that promote healthier lives, even in times of crisis and complexity (Kimhi, Goroshit, and Eshel 2013; Perdomo, Sánchez, and Blanco 2021; Cardinali et al. 2024).

This research adopts a theoretical perspective of community building and social capital to examine its findings. It describes the challenges of displacement into temporary housing shelters, focusing on a case study where over 250,000 residents experienced emergency relocation into 434 evacuation centers during wartime. It outlines a community building intervention in shelters where fragmented and diverse communities faced a new reality of shared living, proposing strategies to enhance community resilience and capacity to cope with the emergency displacement.

This research focuses on the need to build a new shelter community for those displaced to temporary housing. Although there are many challenges associated with displacement to evacuation centers, our research points to two main challenges. First, a weakened social capital due to forced placement of diverse and fragmented communities into densely populated shelters. Secondly, the challenge of maintaining positive momentum over time within the new shelter community. Findings show that despite challenges, living in a shelter community provides vulnerable and isolated populations with opportunities to experience accessible supportive communal life. Reintegration into their original communities should consider that this advantage may become a risk for these populations upon return home, necessitating crisis prevention plans that address this transition. Alongside these challenges, the research identified three leading intervention principles for building a transition community: establishing an emergency shelter team and developing local leadership, strengthening connections with and within the diverse community to create social cohesion, and activating the community in adapted ways while renewing life routines.

The professional intervention principles identified in the research correspond with community building processes described in the literature (e.g., Chaskin et al. 2006; Douglas et al. 2016; Itzhaky and Bustin 2018; Minkler 2012). Community intervention in shelter facilities addressed several key areas, including identifying and harnessing local capacities, establishing community structures to serve the unique needs of the evacuees, strengthening social capital by promoting inclusive and large participation, and empowering local leadership among the displaced, despite the chaotic situation. In the research, as in the literature (Aldrich and Meyer 2015; Binder et al. 2023; Scannell et al. 2016), the success of interventions relies on strengthening social capital.

The research found that when conditions allowed groups from different backgrounds to work together, mutual influence and opportunity for connection were evident. As part of the shelter community, professionals needed to employ bridging methods, such as dialog that breaks down prejudices. The discourse in organized social gatherings at the evacuation center often aimed to dismantle tensions and foster direct communication between the displaced residents. When connections between sub‐communities were built systemically, they contributed to building a shelter community centered around their shared reality. The shelter communities were built out of fragmented and split home communities. Some evacuees came from socially cohesive and intact communities, exemplifying bonding social capital. Others comprised a diverse group of individuals inexperienced in communal life and reliant on external support, exemplifying bridging social capital. As noted by Neal (2015), bonding social capital and bridging social capital can develop together under certain ecological and behavioral conditions, enhancing the collective assets of both (Hughey and Speer 2002). Therefore, while community building in emergency facilities presents challenges for all displaced persons, it also unveils empowering social opportunities. The embodiment of community empowerment enables those closest to the problem to be part of its solution (Christens, Inzeo, and Faust 2014; Stahlhut and Bowes 2003), and even to grow and develop from joining new partners.

This research supports evidence from previous studies indicating the importance of prompt intervention in rebuilding life routines after a disaster (Helly, Efrat, and Yosef 2021; Scannell et al. 2016). In addition to restoring essential formal routines such as employment and education, community building interventions in evacuation centers promote functional continuity by forming communal structures and mobilizing the community around them. This includes shelter emergency teams, need‐based taskforces, assemblies for all residents in the evacuation center, activities for diverse population groups and ages, group therapy, and relief events. Molitor et al. (2011) note the positive association between community events and strengthening social capital. These events connect people, foster new relationships, and increase trust. For disaster survivors and displaced persons, group meetings, whether recreational or dynamic, help cope with emotional distress and improve well‐being and recovery (Huang and Wong 2013).

Amidst the many challenges and uncertainty of displacement, the formation of a unified community from diverse sub‐communities is a testament to resilience and hope. In this context, Cox and Perry (2011) note that the loss of place among displaced persons after a disaster is accompanied by disorientation, which can persist and profoundly affect their health and well‐being. Therefore, the aspiration for a rapid return to normal life as much as possible, is not only logical but necessary for healthy functioning. The findings of the current study support the claim that just as a positive and strong attachment to place is formed through life routines within a community, emergency interventions should aim to create or restore such routines in the new transition community, where familiar routines do not exist. As temporary evacuation often lacks a clear end date, establishing life routines can help mitigate uncertainty by providing a predictable structure for day‐to‐day life and longer‐term activities, such as attending school and work. Life routines provide individuals with a sense of security due to an existential continuity they offer in relation to time and space. Therefore, it is similarly essential to allow for similar continuity in the rehabilitation of displaced persons (Helly, Efrat, and Yosef 2021).

The limitation of this research is that the knowledge was primarily gathered from professionals working with displaced individuals, but it does not directly include perspectives from the displaced populations. While many research participants are displaced themselves and reside in shelters like others, the research predominantly presents a professional point of view. This article contributes to practice, policy, and research on displacement as a global phenomenon by describing the main challenges of community building in shelters, and detailing principles of communal intervention, their process, and operation. The presented intervention in this study may help alleviate some of the severe implications of collective traumas. While some experiences may be influenced by cultural differences, the Israeli case study reflects the broader multiculturalism seen in Western societies. It exemplifies universal practices such as democratic participation, organized support from authorities, and interventions alongside diverse, modern social networks. Thus, the findings are applicable to other contexts with similar social structures and community dynamics.

Finally, while this research supports studies that advocate for the evacuation of groups and communities as organic units (Aldrich 2011; Benhura and Naidu 2021; Ma et al. 2013), it also urges policymakers to adopt preparedness strategies that foster cross‐community and cross‐population relations during routine times. Connecting diverse populations through joint actions facilitates community building and strengthens social capital (Neal 2015). Often, residents of neighboring settlements are merely estranged neighbors with no association beyond the shared use of public spaces. However, a disaster that strikes the area does not distinguish between community or social boundaries. This research reinforced the argument that building bridges between different groups and communities during times of routine will enhance their resilience in future disasters (Aldrich and Meyer 2015) and promote embracing diversity through increased intercommunity collaborations.

As this study was conducted among social workers who frequently encounter the adverse effects of social disparities and fragmentation, this study offers findings of particular relevance to community social work policy and practice. These findings highlight opportunities for social change, addressing both immediate challenges and long‐term structural issues. Immediate interventions aim to enhance welfare and wellbeing beyond the provision of safe temporary housing by building a new shelter community that fosters connections among diverse and fragmented groups. In the long‐term, the findings emphasize the importance of promoting resilience by establishing cross‐community bridges during routine times. Strengthening isolated communities as if they were islands within society is insufficient; instead, authentic bridges must be built between diverse communities to enhance cohesion, mutual respect, and understanding—essential elements for cultivating strong social capital on a broader societal scale. Thereby, split communities may be better prepared for shared living with other diverse and fragmented communities in future evacuations.

## 5 Conclusion

This study contributes to understanding the role of community building in disaster recovery and offers three key recommendations for practice and policy. First, beyond securing safe temporary housing, a vital part of recovery involves community building among displaced residents. Second, while safety during relocation is paramount, our findings indicate that emergency evacuations often lead to the separation of communities across multiple shelters. Therefore, it is essential to form a new shelter community that fosters connections between diverse and fragmented groups. Third, our research emphasizes the importance of building cross‐community bridges not only during emergencies but also during routine times to reduce tensions and estrangement in future evacuation to shared living arrangements. Overall, this study outlines challenges faced by displaced communities in temporary housing and proposes a community building strategy that emphasizes empowerment interventions, strengthening social capital, and cultivating a cohesive community rooted in shared experiences, ultimately supporting long‐term recovery and resilience.

## Author Contributions

We confirm that both authors contributed equally and significantly to all stages of this research, including data collection, analysis, and writing of the final article. Both authors are equally accountable for the work undertaken.

## Ethics Statement

We have complied with APA ethical principles in treatment of individuals participating in the research described in the manuscript. The research was approved by the Institutional Review Board (IRB) at the University of Haifa responsible for the protection of human participants. Throughout all stages of research, we maintained meticulous adherence to ethical obligations.

## Conflicts of Interest

The authors declare no conflicts of interest.

## Data Availability

We confirm that all data obtained from the subject research is present and available for the use of the authors. As data is sensitive and personal it is not publicly available due to confidentiality, however, some anonymous supporting data may be available upon reasonable request from the corresponding author. As the corresponding author, I confirm that I have liaised with the co‐author of this article on all declarations and disclosures herein.
